# An algorithm to create model file for Partially Observable Markov Decision Process for mobile robot path planning

**DOI:** 10.1016/j.mex.2024.102552

**Published:** 2024-01-11

**Authors:** Shripad V. Deshpande, R. Harikrishnan, Jahariah Sampe, Abhimanyu Patwa

**Affiliations:** a Symbiosis Institute of Technology, Pune Campus, Symbiosis International (Deemed University), Pune, India; b Institute of Microengineering and Nanoelectronics, Universiti Kebangsaan Malaysia, Bangi, Selangor, Malaysia

**Keywords:** POMDP, Uncertainty, Mobile robot, Probabilistic technique, Obstacle avoidance, Path planning, PCMRPP

## Abstract

The Partially Observable Markov Decision Process (POMDP), a mathematical framework for decision-making in uncertain environments suffers from the curse of dimensionality. There are various methods that can handle huge sizes of POMDP matrices to create approximate solutions, but no serious effort has been reported to effectively control the size of the POMDP matrices. Manually creating the high-dimension matrices of a POMDP model is a cumbersome and sometimes even impossible task. The PCMRPP (POMDP file Creator for Mobile Robot Path Planning) software package implements a novel algorithm to programmatically generate these matrices such that:

• The sizes of the matrices can be controlled by configuring the granularity of discretization of the components of the state and
• The sparseness of the matrices can be controlled by configuring the spread of the observation probability distribution.

This kind of flexibility allows one to achieve a trade-off between time complexity and the level of robustness of the POMDP solution.

## Graphical abstract

**Figure:**
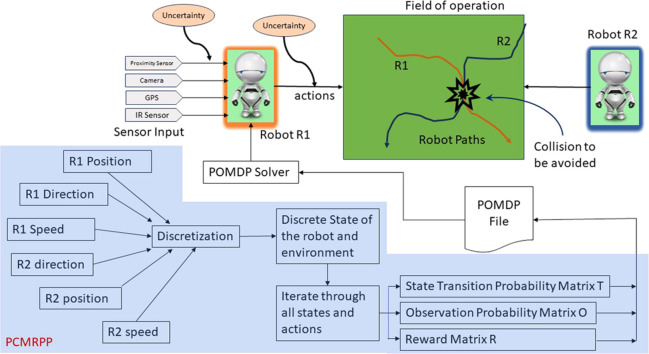

Specifications table

**Table:** 

Subject area:	Engineering
More specific subject area:	Robotics path planning, Uncertainty handling of robotic states.
Name of your method:	PCMRPP.
Name and reference of original method:	POMDP.
Resource availability:	https://data.mendeley.com/datasets/jwdvxscr8n/1

**Method details**

## Introduction

A Mobile Robot System (MRS) consists of a set of mobile robots, with each robot assigned a certain task (e.g., material handling in a manufacturing or assembly sector). These robots are expected to have certain functionalities namely: mapping of the surroundings, locating itself in the map (position and orientation) and searching for the optimal path to the target destination with collision avoidance. Out of these functionalities, path planning turns out to be the most challenging one because of the exponential increase in complexity with the increase in the number of robots.

The robot's path planning strategy comes from methodical handling of the uncertainty that is invariably part of any information processed by the robot. The robot senses the environment using sensors and uses observations to conclude its present state. The uncertainty in robot's path planning could be at sensor level, at communication level or at robot's action level. The uncertainty errors make the robot's perception of its state non-deterministic [1], thereby creating a situation where either a wrong path could be generated or even a collision could be possible. Hence handling uncertainty is very crucial. Many path planning algorithms [2], [3], [4], [5] are developed based on the use case scenarios such as finding the optimal path (minimum turns, shortest, fastest) as well as considering the dynamic nature of the environment.

POMDP has been a well-proven framework for handling uncertainty using probabilistic techniques. It allows the robot to perceive the state of the environment as a probability distribution over multiple states called belief states. POMDP based solutions are proposed for estimation and selection strategies on discrete as well as continuous observation spaces [6]. POMDP serves as the underlying framework for search strategies in 2- dimensional as well as 3-dimensional space [7]. In summary, POMDP acts as a generalized method for the mobile robots acting under uncertain conditions and provides a means for probabilistic reasoning to create a decision-making strategy.

### POMDP

**Fig. 1:**
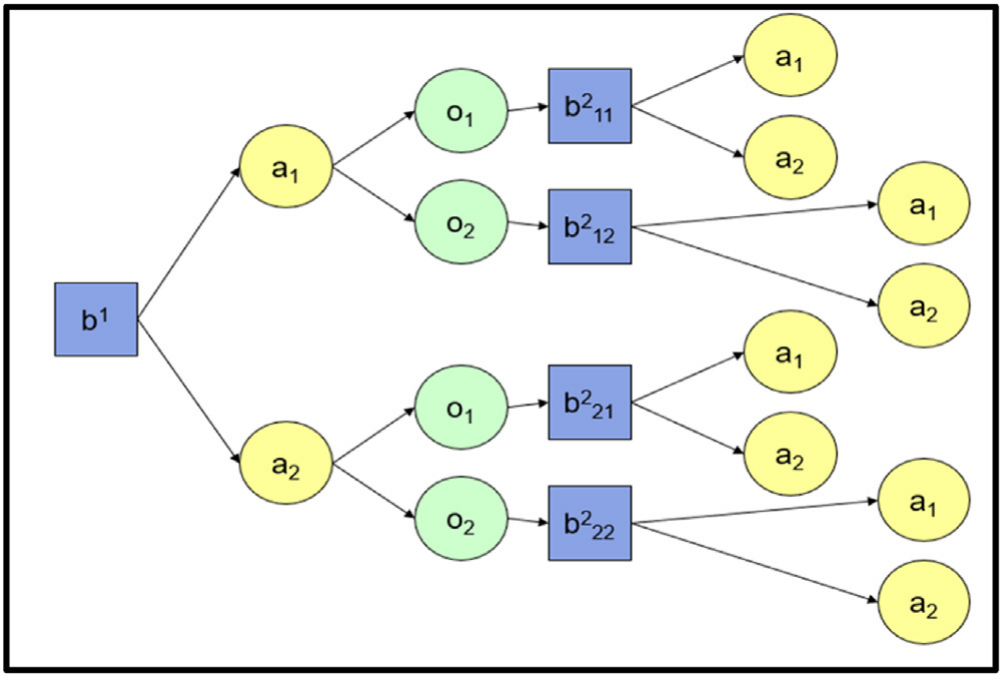
Decision tree representation of POMDP.

Fig. 1 illustrates a decision tree for a POMDP policy. In this figure, “b” is the belief state with its upper suffix indicating the time horizon, first lower suffix is the action no of previous state and second lower suffix the observation. “a” is one of the actions in the action-space. “o” is one of the observations in the observation space. There is an additional step in the process of POMDP compared to Markov Decision Process (MDP). Instead of observing a state and performing an action, first an observation is made and then the belief state is calculated, and finally an action is chosen.

**Fig. 2:**
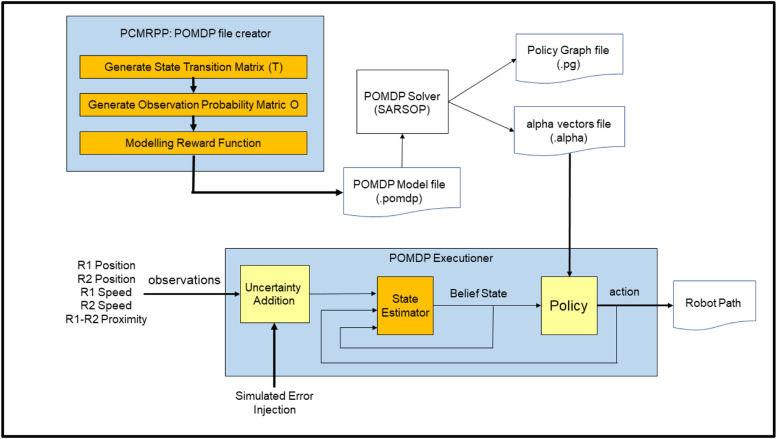
Creation and implementation of a POMDP file.

For creating a POMDP file, the initial requirement is to generate a state transition matrix (T) and an observation probability matrix (O), and a reward function (R) (Refer to Fig. 2). A text file in .POMDP format mentioning the States, Observations, Actions, Transformation matrix, Observation matrix and Rewards matrix should be created. Furthermore, a solver is required to solve the POMDP text file wherein, any solution algorithm could be chosen by the user. The solver will give the output in two file formats, .alpha file will give the value function for every state and action. The .pg file will give the policy graph that could further be used as input for a scenario coded in a visualisation tool.

The POMDP model is constructed using a 7-component tuple {S, A, Z, T, O, R, *γ*} [8], where S, A and Z are the sets of states, actions, and observations respectively. The probability of the robot being in state *s*_*t+1*_ after taking action *a*_*t*_ at time *t* in state *s*_*t*_ is modelled as Transformation matrix T as given in Eq. (1).

(1) $$\begin{array}{c} T\;\left(s_{t}\;a_{t}\;s_{t+1}\right)=P\;\left(s_{t}\;a_{t}\right) \end{array}$$

*O* is the observation matrix indicating the probability of the robot receiving observation after it takes action in state *s_t_*. Refer Eq. (2).

(2) $$O\;\left(s_{t}a_{t}o_{t}\right)=P\left(s_{t}a_{t}\right)$$

*R* is a reward function indicating a positive or negative reward the robot gets for an action in a specific state. The action taken by the robot is for immediate as well as future gains [9]. This is modelled by *γ*, the discount factor.

The size of the T and O matrix is ∏(|S|,|Z|,|A|)and the sparseness of both the matrices depends on the probability spread of the belief state. Creating these matrices manually is too difficult or impossible. Hence, a software package, PCMRPP, is proposed for programmatically generating the POMDP model file based on two critical options configured by the user - the granularity of discretization and the span of the probability distribution.

A POMDP solver tool converts the POMDP model into a policy. There have been various algorithms for the solver ranging from the initial exact solution [8] to approximate solution methods like [10,11], and more recently [12]. Some algorithms use heuristic methods to arrive at a fast approximate solution [5]. A class of algorithms called point-based value iteration have the potential of going beyond discrete space and reaching continuous space POMDP [13]. There are also online solvers [14] and offline solvers [6] which give approximate solutions for the POMDP. Even though these algorithms focus on speeding up the solving of the POMDP model, there have been very few attempts to automate the process of generating the POMDP model file itself.

For a reasonably accurate solution of POMDP, the sizes of *S* and *Z* can easily cross a few thousand. This will create large-sized T and O matrices. The time complexity of solving the POMDP depends on the sizes of these matrices [7] and how sparse they are. The software package PCMRPP allows the user to control the sizes of the T and O matrices by controlling the granularity of discretization of the robot parameters as well as sparseness of the O matrix by controlling the belief state's probability distribution span. The POMDP solver takes in the POMDP model and generates either a set of *alpha vectors, a policy graph,* or both [15]. These *alpha vectors* are used in a dot-product with the belief state to decide the most optimal action the robot must take. Hence, the probability spread of the belief state decides how fast the dot-product is computed and consequently how fast an action is decided during run-time.

Efficiently solving POMDPs presents two significant challenges: the curse of history and the curse of dimensionality [16]. Historically, even relatively small real-world POMDPs with fewer than 30 states created challenging computational complexity, with the best solvers requiring hours to several days to converge. Such prolonged computation times render POMDPs unsuitable for practical applications in real-life robotic systems. Consequently, the need for enhanced robustness and efficiency became paramount. To address this time complexity issue, various approximate POMDP solvers have emerged, including techniques like the Belief Compression method [14] and Value Function approximation [6]. These approaches aim to mitigate the computational demands associated with POMDPs, making them more feasible for real-world robotic challenges.

In the last 20 years, sampling-based solvers have been tried successfully, opening doors to many efficient action strategies for POMDP problems. Although these methods do not compute optimal strategy, they are often sufficient to improve robustness substantially. These solvers come in two forms. Online solvers, e.g., [17], etc., do not take into account historical states but compute the best action based only on the latest belief state. This is done for every time step. These solvers have the advantage of handling large POMDPs [18], but they have the drawback of being slow compared to offline solvers [7]. The Offline solvers can arrive at better policies than online solvers for the sampled belief states, but usually, they fail to scale up to large POMDP [19]. Recently there have also been successful attempts to use Deep Reinforcement Learning techniques for solving POMDP [19].

In this study, we have developed an algorithm that is helpful in generating a Partially Observable Markov Decision Process (POMDP) model for a 2-robot system. This algorithm allows the user to control the complexity of the POMDP file, which in turn controls the complexity of resulting policy generated for robot path planning. This finally governs the time taken by the robot hardware to take correct path decisions when executing the policy in run-time. This control over time complexity is the significant aspect of our work. There are two key methods for determining the time complexity of the final policy that will be executed on the robot in real-time:

1. Selecting the coarseness of discretization.
2. Choosing the number of probability entries per action-state row.

This allows one to trade off the robustness of handling uncertainty versus the execution time and allows one to control it minutely. In this work, a POMDP based path planning strategy is implemented in a three-step process. In the first step, the proposed methodology is used to generate a POMDP model file. The SARSOP implementation of the POMDP solver [5] is used for the second step. And in the third step, the resulting policy is executed in a simulated environment of two mobile robots moving on a collision course. This execution is done by a software program called “POMDP Executioner” (Refer Fig. 2). This program is not part of the POMDP generation algorithm proposed in this research work. The testing is done by varying the span of the probability distribution, and the results are validated. The remainder of the paper talks about how the methodology is implemented. The last part of the paper shows the validation of the proposed method based on various scenarios.

## Methodology

### States and actions formulation

**Fig. 3:**
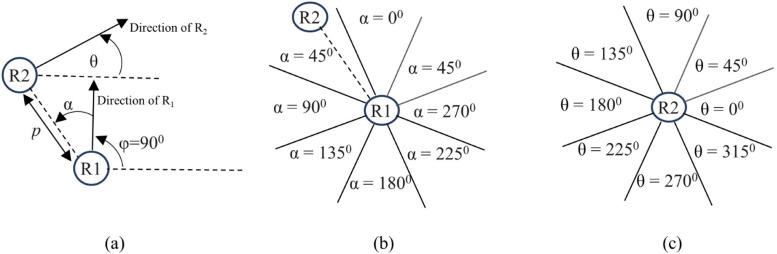
(a) Parameters of the state definition. (b) Discretization of *α.* (c) Discretization of *θ.*

Two mobile robots are assumed to move in a 2-D bounded field without static obstacles. Each robot calls itself *R_1,_* and the other robot as *R_2_*. Refer to Fig. 3. Each robot fixes the frame of reference by always assuming its own direction straight northward(φ=90^°^). The robot does all computations with respect to this frame of reference. The state of the robot can be formulated using a combination of parameters. Some parameters are fully deterministic, whereas some are uncertain [20], leading to a state with mixed observability. In this research work, the state of the robot is modelled using five parameters (Refer Fig. 3(a)) – its speed (*r1s*), the angular direction of the other robot (*α*), the direction in which the other robot is moving (*θ*), the speed of the other robot (*r2s*) and the distance between the two robots (*p*).

For a typical configuration, the parameters α and θ are discretized into 8 sectors, as shown in Fig. 3(b) and (c). The proximity parameter *p* is discretized into 3 levels-No-proximity, Close-proximity, and Collision. The speeds of the two robots are discretized into low, medium, and high. One combination of each of the parameters is one unique state of the robot. The angle through which the robot can turn is discretized to a level that can be configured. For example, a typical configuration of the turning angle being in multiples of 45° will result in 6 actions - turn right 90°, turn right 45°, go straight ahead, turn left 45°, turn left 90° and the “Stop” action.

### About PCMRPP package

**Fig. 4:**
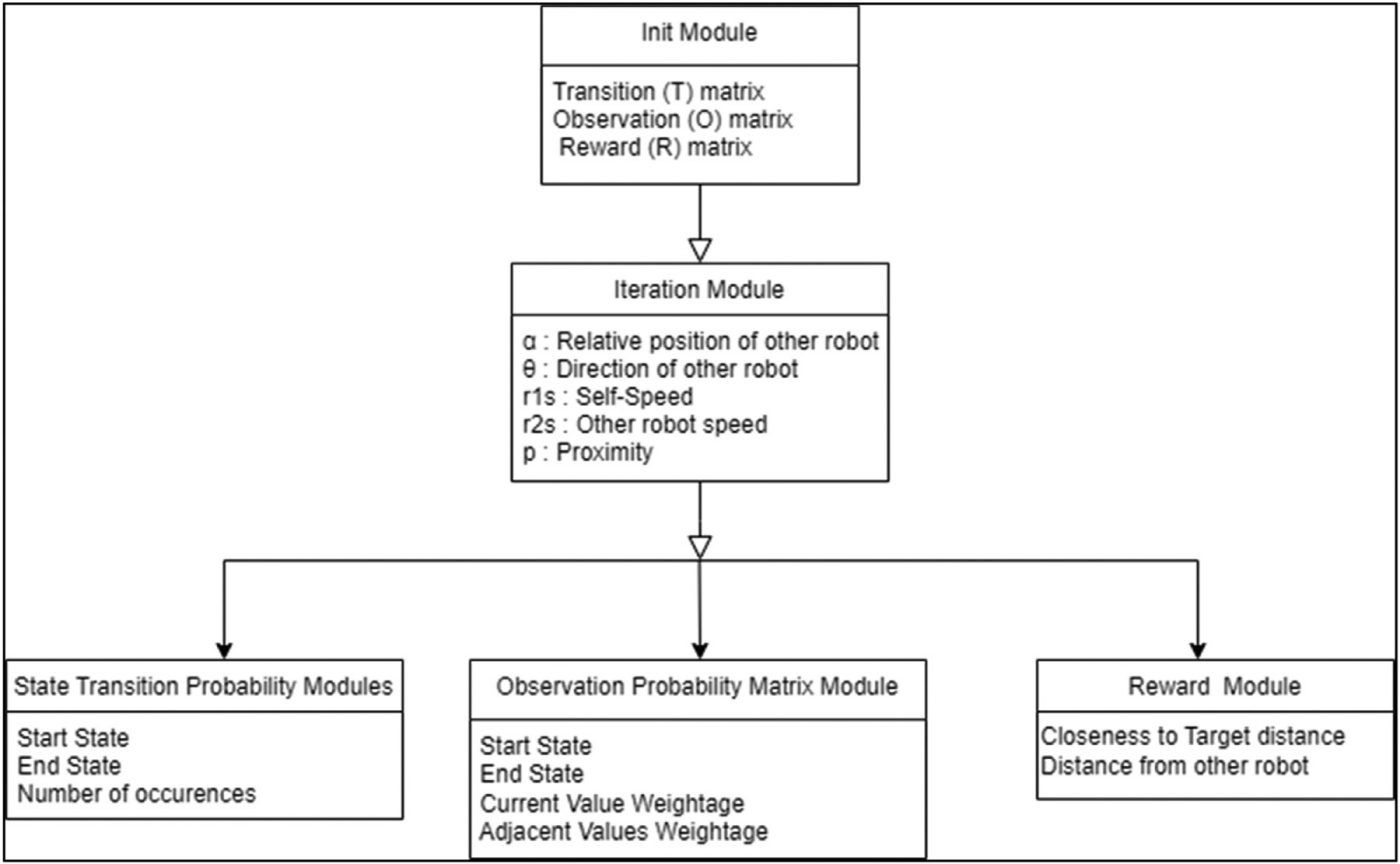
Modules in PCMRPP software package.

PCMRPP algorithm is realized as a software package. It is written in C and implemented for Linux environment. It is constructed as a combination of 5 software modules. Refer Fig. 4.

### Key data structures

Refer Fig. 6.

**Fig. 5:**
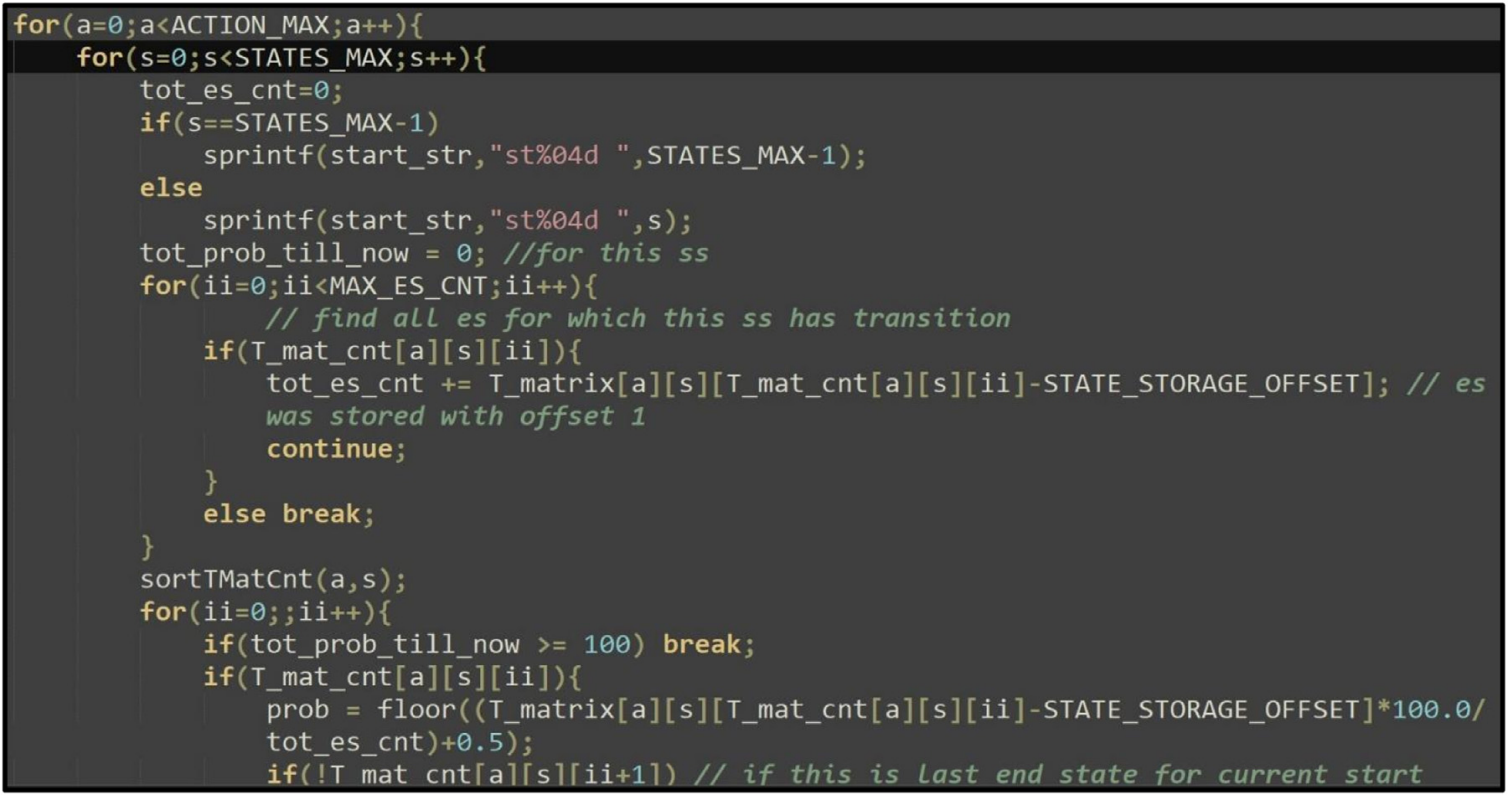
Code snippet of State Transition Probability computation.

**Fig. 6:**
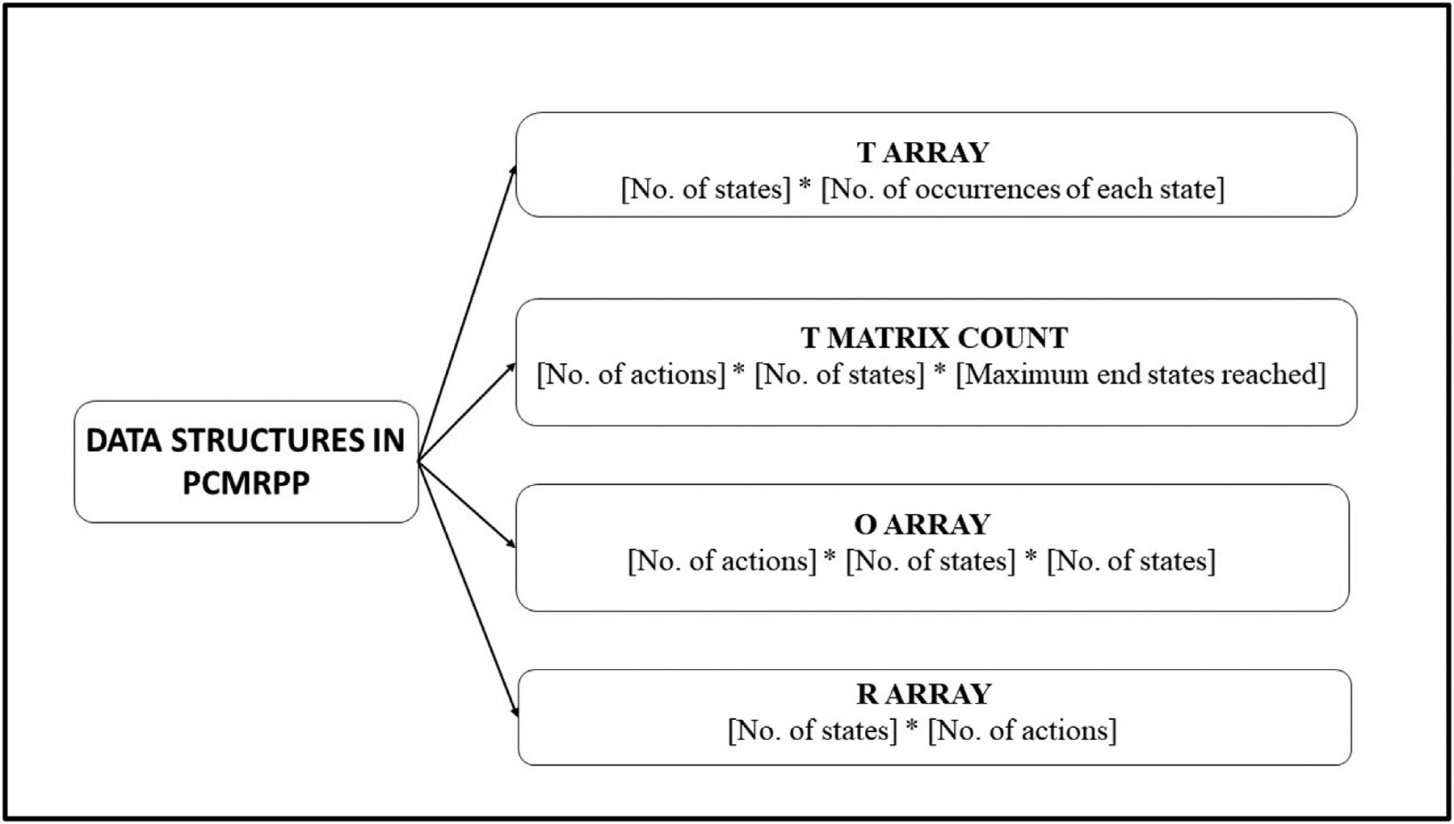
Key Data structures in PCMRPP.

T Array: *T*[*noStates*][*noOccurances*] – This array stores the number of times an end state is reached from a start state because of an action. This is used for Transition probability computation.

**Fig. 7:**
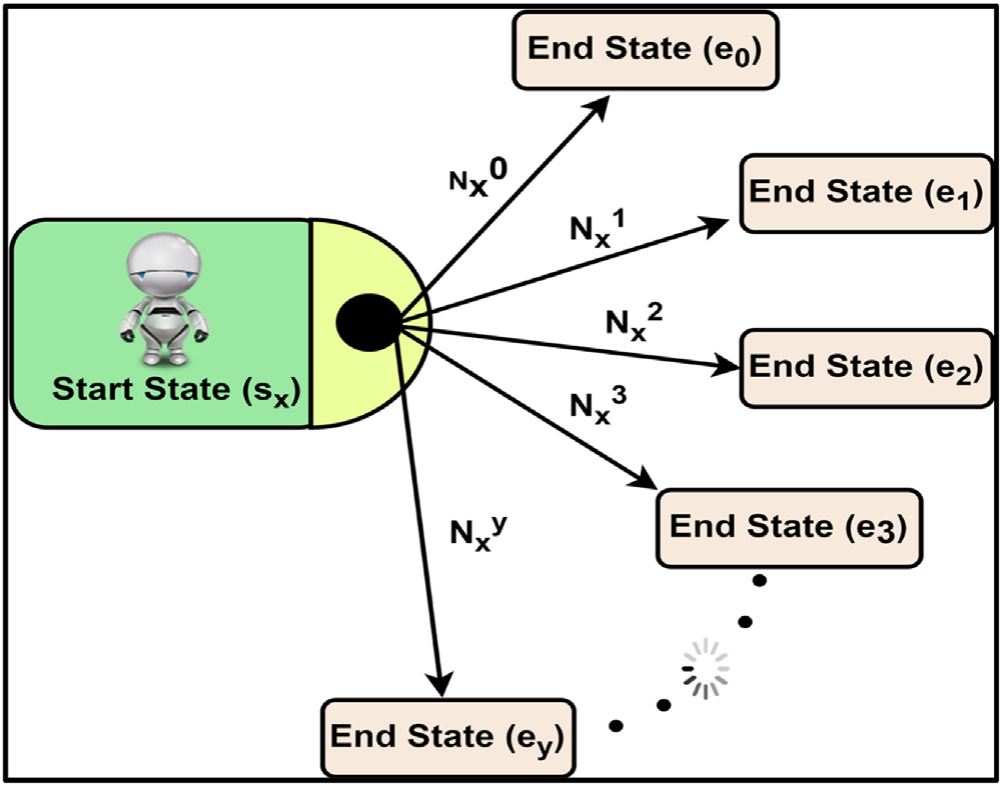
Calculating state transition probabilities in T Matrix.

T Matrix Count: T_mat_cnt[*no*A*ctions*][noStates][max_es_cnt] – This array is used for recording the transition from each start state to multiple end states as a result of a specific action. Eq. (3) works on this array. Refer Fig. 7.

O Array: O[*no*A*ctions*][noStates][noStates] – This array is used for Observation probability calculation by accumulating weightages of two neighbouring values for each of the 5 parameters (α, θ, r1s, r2s and p) constituting the state. Eq. (4) works on this array.

R Array: R[*no*S*tates*][noActions] – This is the array for storing the rewards for each action and state.

Table 1 describes how the individual modules of PCMRPP are developed. These modules play their independent parts while generating a POMDP file and allows users to gain control over the time complexity during creation of the file.

**Table 1:** Functionalities of the modules in PCMRPP.

Module name	Functionality
**Init** module	Initializes the various data structures, like Transition Matrix, Observation matrix, etc., with basic parameters.
**Iteration** module	Iterates through all the values of the five parameters - *α, θ, r1s, r2s*, and *p* in discrete steps. In each iteration, from *t* to *t* *+* *1,* the robots’ positions are updated as per present pose, action, and speed. The new values of all the parameters are discretized and the new state is formulated. For each start state *s_x_*, an array records all the end states *e_y_* that are reached and how many times it is reached $\left(N_{x}^{y}\right)$.
**State Transition Probability** module	Works on the record of start and end states created by the iteration block and calculates the State Transition Probability *T* by Eq. (3). The start state *s_x_* transitions into end state *e_y_*, and the number of occurrences of the transition $T(s_{x},\;e_{y})$ is $N_{x}^{y}$. The code snippet which implements this algorithm is shown in Fig. 5. (3) $$Transition\;Probability\;T\left(s_{x},\;e_{y}\right)=\frac{N_{x}^{y}}{\sum_{a=0}^{y}N_{x}^{a}}\;\left(3\right)$$
**Observation Probability** module	Creates the O matrix. Each of the five parameters of the robot is given weightage such that the current value of the parameter has 80 % weightage and the adjacent values on both sides have 10 % weightage. For example, if α is in sector 3 (90° ≤ α ≤135°) then α being in sector 3 is assigned 80 % weightage, α being in sector 1 (45° ≤ α ≤ 90°) and sector 4 (135° ≤ α ≤ 180°) each given 10 % weightage. The same process is repeated for other parameters. One combination of all parameters having non-zero weightage values give one observation *o_y_* and the addition of the corresponding weightages is denoted by *w_y_*. The observation probability is then computed as in Eq. (4). In this equation, the upper suffix of the weight “w” indicates component of the state and lower suffix indicates the weight 0,1 or 2 based on neighboring states. The weights are chosen empirically. (4) $Observation\;Probability\;\left(s_{x},\;e_{y}\right)=o_{y}=\;\frac{w_{y}}{\sum_{i,j,k,l,m=1}^{3}\left(w_{i}^{\alpha }+w_{j}^{\theta }+w_{k}^{r1s}+w_{l}^{r2s}+w_{m}^{p}\right)}\;\left(4\right)$ With this, a list of observation probabilities is obtained for an end state e_y_. The probabilities in this array are then sorted in ascending order and only first *M* values in this array (M being the configured value for probability spread) are picked up for further processing, discarding the rest.
**Reward** module	Creates the reward matrix. The rewards and penalty values are decided based on closeness to convergence. If current action takes the robot towards its goal, it gets proportionately high reward and if it takes the robot away from the goal, it gets proportionate penalty. These reward and penalty values are not configurable. But of course, they can be modified by editing the code and rebuilding. The robot gets a positive reward on - reduction of distance to the goal, increasing distance from another robot, turning away from another robot, turning towards the goal, etc. The robot gets a negative reward for – collision, moving towards a potential collision, going away from the goal, etc.
**Utility functions** module	Collection of functions like parsing command line options, discretization functions, distance finding function, etc. used by other modules.

### Method validation

A few illustrative examples are provided with a few typical configurations. The POMDP file is created in the format given in [20]. For these examples, discretization levels for all 5 state parameters and actions are decided and the generated pomdp file is used as input to a solver. The number of non-zero entries of T, O and R matrices is recorded. The sparseness of the matrix is calculated by Eq. (5).

(5) $$Matrix\;sparseness=\;\frac{No.\;of\;non-zero\;entries\;in\;the\;matrix}{Total\;size\;of\;Matrix}$$

**Example 1**:

**Table:** 

α and *θ*	Both discretized into four levels – equally divided from 0° to 360°
*r1s and r2s*	Both discretized into three levels – low, medium, high
p	Discretized into three levels – Close proximity, No proximity, and Collision.
a	Actions discretized into three levels – turn left 90°, turn right 90°, and move straight forward.
The number of states and observations	144 each
Belief state spread	2

**Example 2**:

**Table:** 

α and *θ*	Both discretized into eight levels – equally divided from 0° to 360°
*r1s and r2s*	Both discretized into three levels – low (L), medium (M), high (H)
p	Discretized into three levels – Close proximity (P), No proximity (N), and Collision (C).
a	Actions are discretized into five levels – turn left 45°, turn left 90°, turn right 45°, turn right 90°, and move straight forward.
The number of states and observations	1152 each
Belief state spread	2

**Fig. 8:**
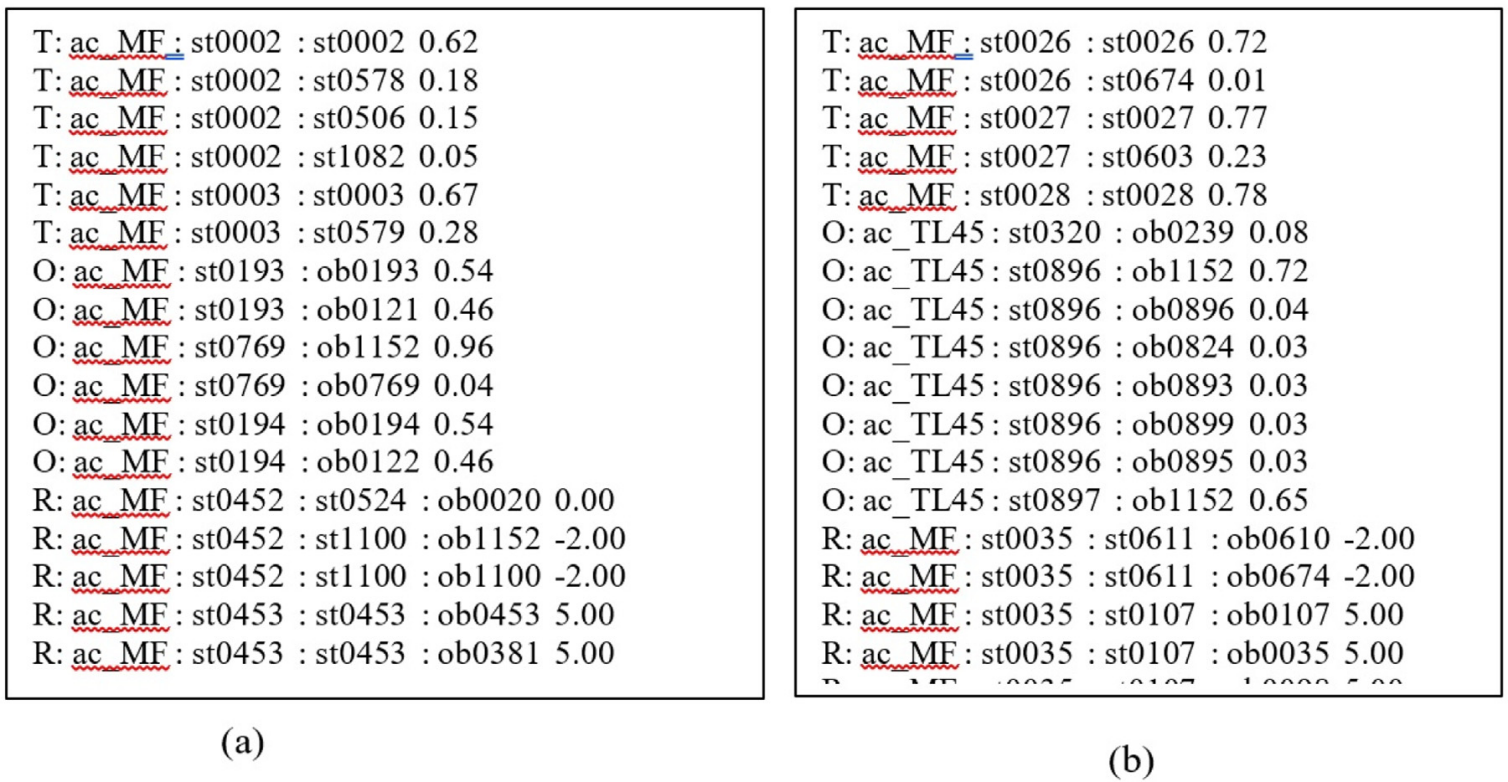
POMDP file snapshot (a) for Example 2 and (b) for Example 3.

The snapshot of the .pomdp file is given in Fig. 8(a).

**Example 3**:

**Table:** 

α and *θ*	Both discretized into eight levels – equally divided from 0° to 360°
*r1s and r2s*	Both discretized into three levels – low (L), medium (M), high (H)
P	Discretized into three levels – Close proximity (P), No proximity (N), and Collision (C).
a	Actions are discretized into five levels – turn left 45°, turn left 90°, turn right 45°, turn right 90°, and move straight forward.
The number of states and observations	1152 each
Belief state spread	10

The snapshot of the .pomdp file is given in Fig. 8(b).

Refer Table 2 for the various configurations used for testing the code. The First 5 columns on the left indicate discretization levels of various parameters. The other columns in the table are N_s_: No. of states and observations; N_a_: No. of actions; d(B): distribution of Belief State; S_T_: Number of distinct T entries in the POMDP file; SP_T_: Sparseness of the T Array (% of non-zero entries); S_O_: Number of distinct O entries in the POMDP file; SP_O_: Sparseness of the O Array (No. of non-zero entries in the O matrix / Total size of the matrix); S_R_: Number of distinct R entries in the POMDP file.

**Table 2:** Example configurations used for generating POMDP files.

α	*θ*	*r1s*	*r2s*	p	N_s_	N_a_	d(B)	S_T_	SP_T_ (%)	S_O_	SP_O_ (%)	S_R_
4	4	3	3	3	288	3	1	5710	2.3	1156	0.46	3754
8	8	3	3	3	1152	5	1	29,854	0.44	9618	0.14	22,936
8	8	3	3	3	1152	5	2	20,484	0.31	20,842	0.31	22,936
8	8	3	3	3	1152	5	10	20,813	0.31	67,884	1.02	67,740

**Fig. 9:**
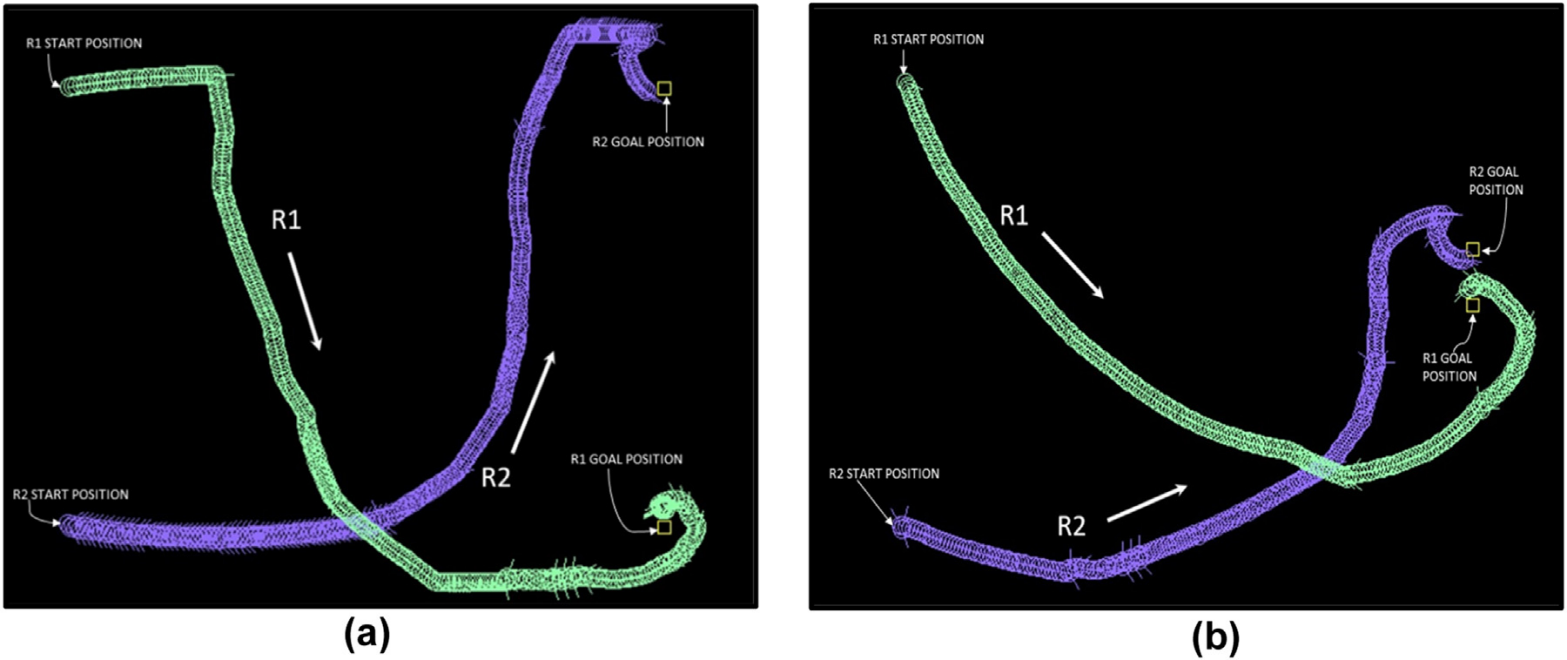
Sample paths generated for two robots. Uncertainty is simulated by adding a percentage error of up to 10 % in the robots’ x and y positions and turning angles.

The POMDP models generated for the above examples are passed through the POMDP solver (APPL Toolkit [5]), and the resultant alpha vectors are used by a POMDP executioner program to generate the robot paths. Robot paths for sample test cases are shown in Fig. 9.

## Conclusion

PCMRPP, is an algorithm to create a POMDP file that serves as a first step to making a policy for a mobile robot path planning in the presence of uncertainty.

This algorithm has the following contributions to the scientific community:

• It allows one to intuitively create a POMDP file with full justification and control over the exact probability figures for each state transition and observation.
• This algorithm has been implemented for a 2-robot system as a console application on Linux platform with standard GCC development environment. It requires no complex installation procedure.
• The software is available in public domain allowing easy customization and thus rapid prototyping and turnaround in the robot path planning exercise. This algorithm can be easily customized for more than two robots, real sensor perception, a more granular level of reward planning, inclusion of static obstacles and support for other platforms e.g., Windows.
• It includes a probability closure logic wherein every probability finally adds up to 1.0 without requiring any clumsy post-adjustment in the model file.
• This research work is tested on limited scenario of two robots but the methodology can be extended to more than 2 robots. The core idea of this work is to control size of the matrices controlling discretization levels of state components. This idea can be extended to more than 2 robots as well.

PCMRPP is distributed as an open-source package, and it is open for contributions and invites users to extend its functionality to make it faster, more robust and more feature-rich.

## Ethics statements

This work neither involved human subjects nor animal experiments. This work also did not involve any data collected from social media platforms.

## Supplementary material *and/or* additional information

• ***Supplementary material***

A document named “Usage of PCMRPP” is included as an appendix with this paper. This document describes briefly the steps detailing how the PCMRPP software can be compiled from source and used.

• ***Additional information***

The format of POMDP file can be found on Anthony Cassandra's [1] original pomdp page: http://pomdp.org/code/pomdp-file-spec.html

## CRediT authorship contribution statement

**Shripad V. Deshpande:** Conceptualization, Methodology, Software. **R. Harikrishnan:** Writing – review & editing, Supervision. **Jahariah Sampe:** Writing – review & editing. **Abhimanyu Patwa:** Data curation, Writing – review & editing, Visualization.

## Declaration of Competing Interest

The authors declare that they have no known competing financial interests or personal relationships that could have appeared to influence the work reported in this paper.

## Data Availability

No data was used for the research described in the article.

## Appendix Supplementary materials

Image, application 1
