# Supplementary material for: An algorithm to create model file for Partially Observable Markov Decision Process for mobile robot path planning
Source: MethodsX. 2024 Jan 11;12:102552. doi: 10.1016/j.mex.2024.102552 (PMC10828799; doi:10.1016/j.mex.2024.102552)
Supplement: Supplementary file 1 [file mmc1.docx]

Appendix

**Usage of PCMRPP Package**

The PCMRPP package assumes following setup of the 2 mobile robots circumventing each other.

**Pre-requisite for pomdp file creation** -

- Linux LTS 20.04
- gcc Compiler

**Steps for Generation of pomdp file**,

1. Download the three files (***pomdpCreateV9-1.c***, ***pomdpV9-1.h*** and ***utility.c***) in a folder.
2. Open pomdpV9-1.h file in a text editor.
3. Modify/visit following lines in this file -
   - #define N_DIV 8

This line controls discretization level of the angles alpha (α) and theta (θ) shown in Fig. 3 (a) in the paper. The number 8 indicates that the angles are discretized to 8 levels so that the value is bounded to any number in the steps of 360/8=45 degrees. If this number is changed to 10 then alpha and theta angles will take values in multiple of 36 degrees. This number must be chosen such than 360 is cleanly divisible by this number.

- - #define R1SPEED_MAX 3
  - #define R1SPEED_MAX 3

These lines control the discretization levels of the speeds of the two robots. The number 3 indicates that the speed is discretized to 3 levels – Slow, Medium and Fast. Modification of this number is generally not advisable since more than 3 levels of discretization does not yield any significant advantage. However, the discretization thresholds can be modified as defined by R_SPEED_S, R_SPEED_M and R_SPEED_F (in the lines 33 to 35). The maximum speed of the robot is assumed as 12. This is hardcoded in the code.

- - #define MAX_OBS 10

This line defines the Observation Probability Spread. The number 10 indicates that the total probability of 100% is spread across maximum 10 states. This controls the sparseness of the O matrix and thereby the number of alpha vectors in final pomdp policy which the pomdp solver gives.

- - #define R_SIZE 15

This line defines the size of robot. Distance between the robots less than this value is taken as collision condition.

- - #define PROXIMITY_MAX 2

This line defines the discretization of proximity level. The number 2 indicates that the proximity is two levels – Close_proximity (next move by either robot towards the other robot will lead to collision) and No_proximity (Safe distance). Please note that apart from these two, a third level of proximity state is hardcoded in the code which is Collision already happened. Since, in this state, other state parameters (like speed of the robots, angles alpha and theta etc) do not matter, it is not allocated a separate identity.

1. Use following command to generate an executable code.

gcc -lm -o pcmrpp pomdpCreateV9-1.c utility.c

1. The executable file “pcmrpp” will be generated in the current directory.
2. For executing the pcmrpp you can give following command line options

*-v n* : Verbosity level (default 0). The number of messages displayed by the program will be more verbose with increasing value of n, but please note that it will take more time for the program to conclude.

-o <file name> : output file name (default mrs.pomdp will be taken)

-h : display help

1. After executing pcmrpp, a .pomdp file will be generated in the same folder.

**Testing of the generated pomdp file**

For testing the pomdp file, a pomdp solver has to be downloaded and used. There are various options for this. One option is <https://pomdp.org/code/> . This is the original pomdp solver by Cassandra [1989]. This solver takes exorbitant amount of time to converge. Another approximate solver is <https://github.com/AdaCompNUS/sarsop>. This generates approximate solution within a reasonable amount of time.

The pomdp solver will create a file called “out.policy” which is a set of alpha vectors in the form of XML. An executioner program written to execute on the robot will read this file and interact with either actual or simulated sensors and generate a path for the robot to follow. For simulation, the sensor values are used as shared variables. For actually executing on robots, the critical state parameters like speed, position, proximity etc must be exchanged between the robots via communication.

**** End of Doc
